# Body Mass and Fat Mass in Refractory Asthma: An Observational 1 Year Follow-Up Study

**DOI:** 10.1155/2010/251758

**Published:** 2010-12-01

**Authors:** Mona Bafadhel, Amisha Singapuri, Sarah Terry, Beverley Hargadon, William Monteiro, Ruth H. Green, Peter H. Bradding, Andrew J. Wardlaw, Ian D. Pavord, Christopher E. Brightling

**Affiliations:** Institute for Lung Health, Clinical Sciences Wing, University Hospitals of Leicester, Groby Road, Leicester LE3 9QP, UK

## Abstract

*Background*. Asthma and obesity are common; however the impact of obesity upon asthma remains uncertain. *Objectives*. To assess relationships between obesity and fat mass with airway inflammation, lung function, and disease control in patients with refractory asthma. *Methods*. 151 refractory asthma patients were characterised for measures of airway inflammation, lung function, Juniper asthma control questionnaire (JACQ), body mass index (BMI), and fat mass index (FMI) derived from dual energy X-ray absorptiometry. Patients were reassessed over 12 months. *Results*. 74% of patients had an elevated BMI. BMI and FMI correlated (*r* = 0.9, *P* < .001). FMI and JACQ correlated in men (*r* = 0.3, *P* = .01). After 12 months 23% lost weight. Weight change over 12 months correlated with FEV_1_ change (*r* = −0.3, *P* = .03), but not with change in JACQ or exacerbations. *Conclusion*. Increased fat mass is common in refractory asthma and is associated with asthma symptom control in men. Loss of weight is associated with improvement in lung function in refractory asthma.

## 1. Introduction

Asthma is common and increasing in prevalence, with an estimated 300 million sufferers worldwide [1]. Severe asthma represents 5%–10% of asthma sufferers and is of particular importance as in this group there is greater morbidity [2] and mortality [3] and an increased burden upon health care resources. Likewise, obesity is increasingly common [4] and is a significant health problem associated with cardiovascular disease, diabetes mellitus and malignancy [5, 6]. There is increasing recognition that severe asthma is a heterogeneous condition; whilst there is an obese predominately noneosinophilic phenotype [7]. Therefore it remains a possibility that obesity may be an important factor in a subgroup of asthmatics. To date studies of obesity in asthma have focussed upon the body mass index (BMI), although a criticism of the BMI is that it does not take into account body composition. The dual energy X-ray absorptiometry (DEXA) scan includes the measurement of regional fat mass and therefore is a more reliable measure of obesity than BMI [8].

Unsurprisingly these two common conditions often coexist, but whether obesity is a major determinant of asthma severity or whether weight reduction impacts on asthma control or exacerbations remains uncertain. We hypothesised that obesity is related to asthma control and that change in weight over 1 year is associated with asthma control and exacerbations. To test this hypothesis, we undertook DEXA scans in patients with refractory asthma and assessed clinical outcomes, including measures of airway inflammation and symptom control with fat mass and body mass. We collected data after 1 year and assessed weight changes with respect to airway inflammation and asthma symptom control.

## 2. Methods

### 2.1. Patients

Severe asthmatics attending the “difficult asthma clinic” at the Glenfield Hospital, Leicester, UK, were invited to enter the study. All patients fulfilled the American Thoracic Society (ATS) criteria for refractory asthma [9]. Patients that had had a severe exacerbation requiring high dose oral corticosteroids and or antibiotics in the last 6 weeks were excluded. The study was approved by the Leicestershire, Northamptonshire and Rutland ethics committee, and all patients gave informed written consent.

### 2.2. Measurements

All patients were assessed using a standardised protocol. Patients underwent spirometry pre- and postbronchodilation with 200 *μ*g salbutamol (Vitalograph, UK), allergen skin prick testing for *dermatophagoides pteronyssinus*, dog, cat, aspergillus, and grass pollen; total IgE; sputum analysis [10], exhaled nitric oxide (measured at 50 ml/s; NIOX, Aerocrine, Sweden) and disease symptom control assessed by the Juniper asthma control questionnaire (JACQ) [11]. JACQ is a standardised questionnaire composing of 7 questions where responses range in score from 0 to 6; 0 is complete control, and 6 is uncontrolled asthma symptoms, including a score for prebronchodilator forced expiratory volume in 1 second (FEV_1_). An average JACQ score of >1.5 is considered to represent poor asthma symptom control [12]. Body habitus was assessed using the body mass index (BMI) [13] and detailed body composition by dual energy X-ray absorptiometry (DEXA scan) (Lunar Prodigy, USA). Fat mass (FM) and fat-free mass (FFM) were analysed from the DEXA scan and were corrected for body surface area to derive the fat mass index (FMI) and fat-free mass index (FFMI). Patients were followed for one year with repeated measures of airway inflammation, lung function, asthma symptom control, and weight. FMI gender differences occur, and there is limited data on normal ranges [8]; we thus chose *a priori* to divide each gender group into tertiles based upon their FMI and assess changes within these groups.

### 2.3. Statistical Analysis

Statistical analysis was performed using PRISM version 5 (GraphPad, San Diego, CA) and SPSS version 16 (SPSS, Inc., Chicago, IL). Parametric data was expressed as mean (SEM), and nonparametric data is expressed as median (IQR). Log-transformed data is presented as geometric mean (95% confidence interval). Correlation was measured using the Pearson correlation (*r*) or Spearman rank correlation (*r*_*s*_) coefficients for parametric and non-parametric data. The coefficient of determination (*R* ^2^) is presented where appropriate. For comparison of unpaired or paired parametric or nonparametric groups, the Student is *t*-test, Paired *t*-test, Mann-Whitney test, and Wilcoxon matched pairs test were used, respectively. For comparison of three groups or more for parametric and nonparametric variables, the one-way analysis of variance (ANOVA) or Kruskal-Wallis test was used and the *χ* ^2^ test for proportions. Significant weight change after 1 year was defined as ±2 kg [14]. Multiple regression was used to assess the relationship of the dependant variable change in asthma symptom control with explanatory (independent) variables using the standard enter method. The independent variables entered into the regression model chosen for clinical relevance were the following continuous variables: (i) change in postbronchodilator FEV_1_% predicted; (ii) change in weight; (iii) change in inhaled corticosteroid dose; (iv) change in sputum eosinophils (log transformed). Collinearity diagnostics were performed and determined that the model showed no violation of multicollinerarity or homoscedasticity, and the residuals observed normality [15]. A *P*  value  of < .05 was deemed statistically significant.

## 3. Results

**Figure 1:**
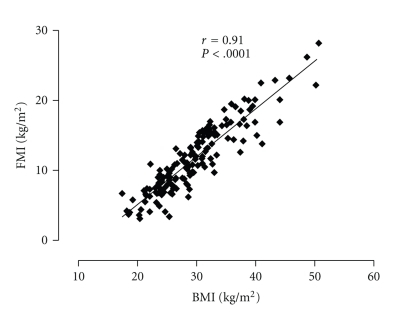
Correlation of fat mass index (FMI) and body mass index (BMI) in refractory asthmatic patients.

151 patients (69 men, 82 women) were entered into the study. Using age- and gender-specific normal ranges [8], we estimated that FMI were increased in 75% of men and 79% of women. FMI and BMI was strongly correlated (*r* = 0.91, *P* < .0001, Figure 1). 2% of patients were underweight (BMI < 18.5), 23% had normal weight (18.5 ≤ BMI < 25), 31% were overweight (25 ≤ BMI < 30), and 44% were obese (BMI ≥ 30). The clinical characteristics for all patients categorised into tertiles based on their baseline FMI are outlined in Table 1. 

We found no difference in BMI, JACQ score or pattern of airway inflammation between men and women. Men had a lower percentage predicted forced expiratory volume in 1 second (FEV_1_) than women (mean difference (95% CI) 8.1% (1.2 to 15.1); *P* = .02) and airflow obstruction (mean (SEM) FEV_1_/FVC% 67 (2) versus 74 (1), *P* < .001). For all patients, there was no correlation between FMI or BMI and exacerbation frequency (*r* = 0.05, *P* = .59; *r* = −0.01, *P* = .88), post-bronchodilator FEV_1_% predicted (*r* = 0.02, *P* = .88; *r* = −0.01, *P* = .91), neutrophilic (*r* = −0.02, *P* = .84; *r* = 0.01, *P* = .92) or eosinophilic airway inflammation (*r*_*s*_ = 0.11, *P* = .22; *r*_*s*_ = 0.08, *P* = .34). There was a statistically significant albeit weak, correlation of FMI and JACQ score (*r* = 0.17, *P* = .04); this relationship was observed in men but not women (*r* = 0.34, *P* = .005; *r* = −0.01, *P* = .93). There was no correlation between JACQ and BMI (*r* = 0.15, *P* = .06). 

**Figure 2:**
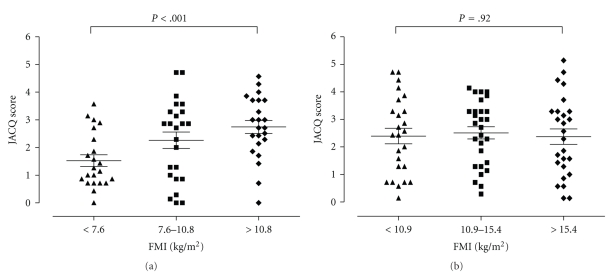
Juniper asthma control questionnaire (JACQ) score after tertile stratification of fat mass index (FMI) for men (top) and women (bottom). Horizontal bars set at mean and standard error of the mean.

After stratification into tertiles, subgroup analysis of men and women revealed that men had a significant difference in JACQ score between the FMI tertiles (ANOVA JACQ and JACQ_(independent of FEV)_ *P* < .001, *P* = .01 Figure 2). Linear regression across the tertiles was also significant (*r* = 0.33; *P* = .005). This difference between tertiles was not evident in women (Figure 2). 

One year follow-up clinic visits with measurement of weight and JACQ scores was available in 129 patients; 8 patients were discharged from clinic, 13 patients were lost to follow up, and 1 patient died. 77% of patients had a change in weight of ±2 kg. 54% of patients had a mean (SD) increase in weight of 4.9 (2.9) kg, and 23% of patients had a mean (SD) decrease in weight 4.4 (2.8) kg. At baseline there were no differences between men and women that subsequently gained, lost or did not have a change in weight. Compared to baseline there was no difference after 1-year follow-up in lung function, maintenance corticosteroid, inhaled corticosteroid dosage, JACQ scores, or eosinophilic airway inflammation in men or women that lost weight, gained weight, or did not change their weight (Tables 2 and 3). 

**Figure 3:**
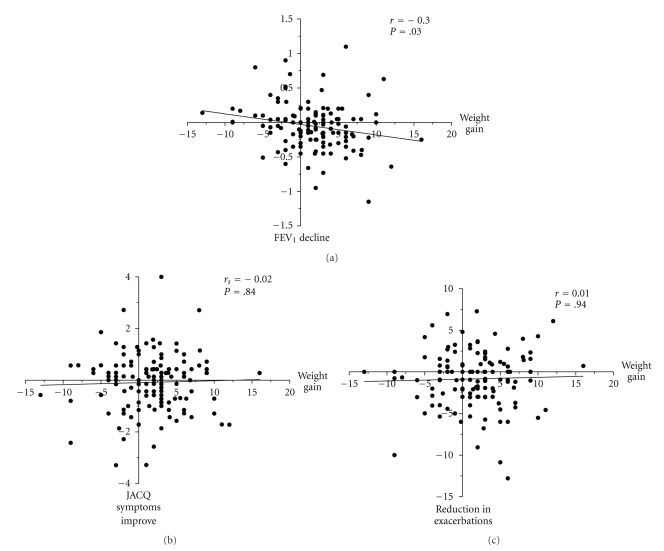
Scatter plot for change in weight versus change in postbronchodilator FEV_1_ (a), JACQ symptoms (b), and exacerbations (c).

Change in weight correlated with change in FEV_1_, but not change in symptoms or exacerbations (*r* = −0.3, *P* = .03; *r* = −0.02, *P* = .84; *r* = 0.01, *P* = .94, resp.) (Figure 3); this correlation was evident in women and not men (*r* = −0.34, *P* = .01 versus *r* = 0.14, *P* = .31). In men but not women, there was an association of change in FEV_1_ and change in exacerbations and JACQ (men: *r* = −0.34, *P* = .01; *r* = −0.46, *P* < .01 and women: *r* = −0.01, *P* = .97; *r* = −0.16, *P* = .20). Multiple regression analysis showed that change in asthma control symptoms after 1 year could not be determined by changes in weight, eosinophilic airway inflammation, lung function, or corticosteroid dosage (*R*_2_ = 0.10, *P* = .13).

## 4. Discussion

We report here for the first time the body composition of a cohort of patients with severe refractory asthma using the DEXA scan. In this group we found that symptom control as measured by the Juniper asthma control questionnaire correlated with fat mass in men, but not women. Fat mass was not associated with lung function, airway inflammation, or exacerbation frequency. Over a 1 year follow-up the majority of patients with refractory asthma attending our specialist asthma clinic gained weight, which was independent of changes in treatment. There were no associations between the change in weight and the change in asthma control, airway inflammation or exacerbations. Interestingly, in women that lost weight there was an improvement in lung function, which was not observed in men. 

The association of obesity and asthma has been evident from epidemiological studies, including case control studies and cross-sectional studies that have shown an increase risk of asthma in obese individuals based on the BMI [16–20], whilst longitudinal cohort studies have shown an increase in asthma in obese patients, but these have often been in self-reported asthma diagnosis [21, 22]. The association between asthma control and obesity is contentious and reports present conflicting findings [23, 24]. These observations have primarily focussed on body mass index as a measure of obesity and more importantly have not focussed on a severe refractory population. We therefore have extended these earlier observations to report for the first time the fat mass, derived from DEXA scans, in patients with refractory asthma. We found a cross-sectional association between fat mass and asthma control in men only. 

We have previously identified a subphenotype of severe asthmatics that were predominately obese non-eosinophilic women [7]. However, we were unable to find any cross-sectional association between fat mass, airway inflammation, and lung function; or between asthma control and fat mass in women. Similarly, we did not find a relationship between obesity and atopy, which has been previously reported by some [25], but not by others [26], suggesting that the cross-sectional associations are complex.

A strength of our findings is that we assessed patients over a 1 year follow-up period. This allowed us to examine the longitudinal relationship between changes in weight and clinical outcomes. Previous studies looking at morbid obesity, mainly in women, and the effect of weight loss following bariatric surgery have shown improvement in asthma control [27]. One-year follow-up of the TENOR cohort of moderate to severe asthmatics found that in the weight reduction group there was a small, albeit significant, attenuation in the exacerbation frequency [14], whilst others have reported that severe exacerbations of asthma requiring hospital admission were associated with longer hospital stay in those with concomitant obesity [28]. Although we did not find any association between weight loss and reductions in exacerbations or asthma control, it did improve lung function, the greatest magnitude found in women, whilst independently in men there was a signal of lung function improvements with asthma control and reduction in exacerbations. Our data suggests that the cross-sectional and longitudinal relationship between gender, obesity, and asthma outcomes are distinct. Importantly, change in weight was not associated with change in airway inflammation or medication. It is possible that weight reduction may reduce the burden on the workload of breathing and increase exercise capacity with the observation of improvements in lung function. The predilection for this improvement in women only may suggest a different fat loss distribution, which may in turn lead to reduction in exacerbations, but needs to be further evaluated. In this study we could not determine a suitable model for change in asthma control over 1 year with respect to change in weight, lung function, and airway inflammation, suggesting that interactions of symptom control in refractory asthma are indeed complex with dissociation in some individuals [7]. 

One potential criticism of our study is that the follow-up assessment did not include a repeat DEXA scan to assess changes in body composition, but relied upon change in weight. However, in this group we found that FMI and BMI were very strongly correlated so we are confident that change in weight was likely to be a consequence of a reduction in fat mass. Although this was an observational study and not a placebo-controlled intervention study, our findings do support a possible association, albeit small between weight reduction and improvements in asthma outcomes and may pave the way for future trials of weight reduction in severe refractory asthma. Importantly, future studies will need to further explore the potential mechanisms of the association between asthma control and obesity, which may have both physical and psychological components.

Taken together this data demonstrates that the relationship between obesity and asthma outcomes in severe refractory asthma is complex. We have identified cross-sectional associations between asthma control and obesity in men, and a longitudinal relationship between weight loss and lung function improvements. These observations need to be replicated in large intervention trials and highlight the potential importance of comorbid obesity in severe asthma.

##  Conflict of Interests

M. Bafadhel, A. Singapuri, S. Terry, W. Monteiro, B. Hargadon, and R. H. Green do not have any financial relationship with a commercial entity that has an interest in the patent of this paper. P. H. Bradding received consulting fees from GlaxoSmithKline. A. J. Wardlaw received grant support from AstraZeneca and GlaxoSmithKline. I. D. Pavord received consulting fees from AstraZeneca and GlaxoSmithKline. C. E. Brightling has received grant support from AstraZeneca, GlaxoSmithKline, and MedImmune.

## Figures and Tables

**Table 1:** Patient demographics.

	Women	Characteristics in women stratified into FMI tertiles				Men	Characteristics in men stratified into FMI tertiles			
		FMI < 10.9	10.9 ≤ FMI < 15.4	FMI > 15.4	*P* Value		FMI < 7.6	7.6 ≤ FMI < 10.9	FMI > 10.9	*P* Value
Number, *n*	82	27	28	27		69	23	23	23	
Age*	46 (19–79)	45 (20–74)	48 (23–79)	48 (19–67)	**0.60**	52 (18–80)	55 (23–78)	52 (23–80)	50 (18–78)	**0.42**
Age of onset of asthma*	20 (2–73)	20 (1–58)	21 (1–73)	21 (1–46)	**0.99**	28 (2–79)	24 (1–58)	31 (1–79)	30 (1–75)	**0.53**
Disease duration yrs	26 (2)	24 (3)	27 (3)	27 (3)	**0.71**	24 (2)	31 (4)	20 (4)	20 (3)	**0.06**
Never smokers %	85	82	89	85	**0.71**	84	78	96	78	**0.18**
Pack year history	4 (1)	6 (3)	3 (1)	3 (1)	**0.39**	6 (2)	6 (2)	3 (2)	9 (6)	**0.39**
Positive atopy status %	68	76	61	69	**0.07**	66	65	77	55	**0.07**
Exacerbation rate in previous yr	4.3 (0.4)	4.5 (0.6)	4.0 (0.6)	4.4 (0.8)	**0.87**	3.5 (0.4)	3.5 (0.7)	3.6 (0.7)	3.3 (0.7)	**0.96**
Maintenance prednisolone %	51	52	54	48	**0.69**	48	52	39	52	**0.10**
Prednisolone dose mg*	11 (5–40)	14 (5–40)	9 (5–15)	12 (5–23)	**0.20**	11 (5–20)	11 (5–20)	13 (5–20)	11 (5–20)	**0.46**
Inhaled corticosteroid %	100	100	100	100	**1.00**	100	100	100	100	**1.00**
Inhaled corticosteroid dose *μ*g${}^{\hat{\;\;}}$	1490 (89)	1427 (150)	1393 (117)	1652 (189)	**0.44**	1547 (86)	1530 (158)	1539 (130)	1648 (84)	**0.83**
Long-acting beta agonist %	90	83	93	92	**0.62**	93	91	91	96	**0.90**
Total IgE (kU/L)	504 (118)	835 (286)	309 (134)	374 (190)	**0.14**	644 (132)	638 (293)	770 (198)	523 (189)	**0.75**
FEV_1_ % predicted^#^	81 (2)	88 (4)	76 (4)	79 (4)	**0.08**	73 (3)	73 (4)	69 (5)	76 (5)	**0.60**
FEV_1_ / FVC^#^ (%)	74 (1)	77 (2)	72 (2)	74 (2)	**0.18**	67 (2)	65 (3)	65 (3)	71 (2)	**0.23**
FE_NO_^ ¶^ (ppb)	32 (24–42)	32 (21–51)	30 (15–58)	33 (20–53)	**0.99**	34 (26–43)	26 (16–41)	37 (21–65)	41 (28–59)	**0.39**
Sputum eosinophil count^¶^ (%)	5 (3–7)	3 (1–9)	6 (3–14)	6 (3–11)	**0.31**	5 (3–7)	5 (3–9)	5 (2–11)	5 (2–10)	**0.72**
Sputum neutrophil count (%)	55 (3)	51 (6)	60 (5)	55 (5)	**0.48**	59 (3)	58 (6)	59 (5)	62 (5)	**0.88**
JACQ score	2.4 (0.1)	2.4 (0.3)	2.5 (0.2)	2.4 (0.3)	**0.92**	2.2 (0.2)	1.5 (0.2)	2.3 (0.3)	2.7 (0.2)	**<0.001**
JACQ score_(independent of _ _FEV_1___)_	2.3 (0.2)	2.4 (0.3)	2.4 (0.2)	2.3 (0.3)	**0.97**	2.0 (0.2)	1.2 (0.2)	2.0 (0.3)	2.7 (0.2)	**0.01**
BMI (kg/m^2^)	30.0 (0.7)	23.2 (0.5)	30.1 (0.6)	36.7 (1.0)	**<0.001**	29 (1)	23.7 (0.6)	27.4 (0.6)	36.0 (1.1)	**<0.001**
FMI (kg/m^2^)	13.3 (0.6)	7.9 (0.4)	13.5 (0.4)	18.6 (0.7)	**<0.001**	10 (0.5)	6.1 (0.3)	8.9 (0.3)	14.2 (0.6)	**<0.001**

Data presented as mean (SEM) unless otherwise stated. *Mean [range]; ${}^{\hat{\;\;}}$BDP equivalent. FEV_1_: forced expiratory volume in 1 second, FVC: forced vital capacity, FE_NO_: fraction of exhaled nitric oxide, JACQ: Juniper asthma control questionnaire score, BMI: body mass index, FMI: fat mass index. Data presented as mean (SEM) unless otherwise stated; ^#^postbronchodilator; ^¶^geometric mean (95% CI).

**Table 2:** Characteristics according to weight change grouping in women.

	Weight loss group *n* = 19			No weight change group *n* = 16			Weight gain group *n* = 31			*P* Value
	Baseline	1 year	Delta Δ	Baseline	1 year	Delta Δ	Baseline	1 year	delta Δ	
Weight, (kg)	81 (4)	76 (5)	−4 (−17 to 9)^†^	74 (4)	74 (4)	0 (−12 to 12)^†^	77 (3)	81 (3)	4 (−5 to 14)^†^	**<0.001**
BMI (kg/m^2^)	31 (2)	29 (2)	−2 (−6 to 3)^†^	30 (2)	30 (2)	0 (−5 to 5)^†^	30 (1)	32 (1)	2 (−2 to 5)^†^	**<0.001**
FEV_1 _% predicted^#^	78 (4)	84 (4)	6 (−6 to 18)^†^	77 (6)	78 (7)	1 (−19 to 20)^†^	83 (4)	81 (4)	−3 (−14 to 8)^†^	**0.51**
FE_NO_ (ppb)^¶^	24.8 (12.9 to 47.7)	25.0 (17.0 to 36.9)	1.0 (0.5 to 2.0)^‡^	44.5 (22.9 to 86.6)	34.9 (19.7 to 61.9)	1.2 (0.6 to 2.9)^‡^	31.8 (21.6 to 46.8)	23.4 (17.0 to 32.3)	−1.4 (−2.2 to 0.8)^‡^	**0.76**
Exacerbation rate	4.5 (0.9)	3.9 (0.8)	−0.6 (−3.0 to 1.8)^†^	3.7 (0.4)	2.3 (0.6)	−1.4 (−2.9 to 0)^†^	3.7 (0.6)	2.7 (0.5)	−1.1 (−2.6 to 0.5)^†^	**0.49**
Maintenance prednisolone, %	47	42		67	67		56	59		**0.11**
Prednisolone dose, mg*	11 (5–40)	9 (3–20)	−2.6 (−11 to 6)^†^	10 (5–20)	9 (5–20)	−1 (−6 to 4)^†^	9 (5–35)	7 (3–25)	−1 (−5 to 3)^†^	**0.96**
Inhaled corticosteroid dose${}^{\hat{\;\;}}$ (ug)	1874 (257)	1889 (111)	15 (−566 to 596)^†^	1325 (121)	1538 (117)	213 (−130 to 555)^†^	1371 (105)	1439 (105)	68 (−229 to 365)^†^	**0.62**
JACQ score	2.6 (0.3)	2.4 (0.3)	−0.2 (−1.0 to 0.6)^†^	2.5 (0.3)	2.3 (0.2)	−0.2 (−0.9 to 0.6)^†^	2.3 (0.2)	2.2 (0.2)	−0.1 (−0.8 to 0.5)^†^	**0.96**
JACQ score_(independent of _ _FEV_1___)_	2.5 (0.3)	2.4 (0.3)	−0.1 (−1.1 to 0.8)^†^	2.3 (0.3)	2.2 (0.2)	−0.1 (−0.9 to 0.6)^†^	2.3 (0.3)	2.1 (0.2)	−0.1 (−0.8 to 0.6)^†^	**0.92**
Sputum neutrophils (%)	44 (6)	65 (5)	20 (4 to 36)^†^	74 (6)	76 (4)	2 (−14 to 18)^†^	51 (4)	69 (5)	18 (4 to 32)^†^	**0.06**
Sputum eosinophils^¶^ (%)	6.5 (2.4 to 17.4)	2.9 (1.2 to 7.0)	−2.2 (−7.9 to 0.9)^‡^	3.4 (1.4 to 8.4)	4.6 (1.4 to 14.9)	0.7 (−2.9 to 0.2)^‡^	6.0 (3.2 to 11.2)	2.6 (1.2 to 5.8)	−2.3 (−6.1 to 0.9)^‡^	**0.01**

BMI: body mass index; FEV_1_: forced expiratory volume in 1 second; FE_NO_: fraction exhaled nitric oxide, JACQ: juniper asthma control questionnaire score; data presented as mean (SEM) unless otherwise stated. ^#^postbronchodilator; ^¶^geometric mean [95% CI];^†^mean (95% CI); ^ ¶^mean [range]; ${}^{\hat{\;\;}}$BDP equivalent; ~median (IQR); ^†^mean difference (95% confidence interval); and ^‡^fold change (95% confidence interval).

**Table 3:** Characteristics according to weight change grouping in men.

	Weight loss group *n* = 11			No weight change group *n* = 14			Weight gain group *n* = 38			*P* Value
	baseline	1 year	Delta Δ	Baseline	1 year	Delta Δ	Baseline	1 year	delta Δ	
Weight, (kg)	83 (5)	78 (4)	−5 (−18 to 8)	86 (5)	86 (5)	0 (−15 to 15)	89 (4)	95 (4)	5 (−6 to 16)	**<0.001**
BMI (kg/m^2^)	28 (2)	27 (1)	−2 (−6 to 3)	29 (2)	29 (2)	0 (−5 to 5)	29 (1)	31 (1)	2 (−1 to 5)	**<0.001**
FEV_1_ % predicted^#^	80 (6)	85 (7)	5 (−15 to 24)	72 (5)	72 (5)	0 (−15 to 15)	71 (4)	73 (4)	2 (−10 to 14)	**0.32**
FE_NO_ (ppb)^ ¶^	19.3 (14.2 to 26.3)	25.8 (16.1 to 41.2)	0.7 (−0.4 to 1.5)	29.4 (17.8 to 48.7)	27.9 (18.4 to 42.5)	−1.1 (−2.0 to 1.8)	41.0 (29.6 to 56.8)	46.7 (35.5 to 61.4)	0.9 (−1.3 to 0.6)	**0.37**
Exacerbation rate	4.2 (1.0)	2.4 (0.8)	−1.8 (−4.5 to 0.8)	2.1 (0.6)	2.5 (0.7)	0.4 (−1.5 to 2.3)	3.9 (0.6)	2.6 (0.4)	−1.3 (−0.7 to 1.3)	**0.78**
Maintenance prednisolone, %										**0.51**
Prednisolone dose, mg*	9 (5 to 20)	14 (5 to 35)	5 (−5 to 13)	8 (5 to 20)	11 (8 to 20)	3 (−4 to 3)	9 (5 to 20)	10 (5 to 35)	1 (−3 to 5)	**0.75**
Inhaled corticosteroid dose${}^{\hat{\;\;}}$ (ug)	1055 (197)	1345 (197)	291 (−289 to 871)	1800 (226)	1800 (226)	0 (−657 to 657)	1558 (110)	1889 (155)	332 (−47 to 710)	**0.14**
JACQ score	1.8 (0.4)	1.9 (0.4)	0.1 (−1.2 to 1.4)	2.5 (0.3)	2.3 (0.3)	−0.2 (−0.5 to 0.2)	2.1 (0.2)	2.1 (0.2)	0.0 (−0.6 to 0.6)	**0.49**
JACQ score_(independent of _ _FEV_1___)_	1.7 (0.4)	1.8 (0.4)	0.1 (−1.2 to 1.4)	2.2 (0.4)	2.1 (0.4)	−0.1 (−1.2 to 0.9)	1.9 (0.2)	1.9 (0.2)	0.0 (−0.7 to 0.6)	**0.48**
Sputum neutrophils (%)	72 (4)	67 (6)	−5 (−21 to 12)	60 (8)	70 (9)	10 (−16 to 36)	57 (4)	72 (4)	15 (4 to 16)	**0.40**
Sputum eosinophils^¶^ (%)	4.0 (1.7 to 9.6)	2.9 (1.1 to 8.2)	−1.4 (−4.8 to 0.4)	4.8 (1.6 to 13.9)	1.5 (0.4 to 5.5)	−3.1 (−14.9 to 0.6)	5.8 (3.3 to 10.5)	3.0 (1.6 to 5.3)	−2.0 (−4.5 to 0.9)	**0.38**

BMI Body mass index; FEV_1_: forced expiratory volume in 1 second; FE_NO_: fraction exhaled nitric oxide; JACQ: juniper asthma control questionnaire score; data presented as mean (SEM) unless otherwise stated. ^#^postbronchodilator; ^¶^geometric mean [95% CI]; ^†^mean (95% CI); ^ ¶^mean [range]; ${}^{\hat{\;\;}}$BDP equivalent; ~median (IQR); ^†^mean difference (95% confidence interval); and ^‡^fold change (95% confidence interval).
